# Fabrication and magnetic properties of granular Co/porous InP nanocomposite materials

**DOI:** 10.1186/1556-276X-6-276

**Published:** 2011-03-31

**Authors:** Tao Zhou, Dandan Cheng, Maojun Zheng, Li Ma, Wenzhong Shen

**Affiliations:** 1 Laboratory of Condensed Matter Spectroscopy and Opto-Electronic Physics, and Key Laboratory of Artificial Structures and Quantum Control (Ministry of Education), Department of Physics, Shanghai Jiao Tong University, Shanghai, 200240, People's Republic of China; 2 School of Chemistry & Chemical Technology, Shanghai Jiao Tong University, Shanghai, 200240, People's Republic of China

## Abstract

A novel Co/InP magnetic semiconductor nanocomposite was fabricated by electrodeposition magnetic Co nanoparticles into *n*-type porous InP templates in ethanol solution of cobalt chloride. The content or particle size of Co particles embedded in porous InP increased with increasing deposition time. Co particles had uniform distribution over pore sidewall surface of InP template, which was different from that of ceramic template and may open up new branch of fabrication of nanocomposites. The magnetism of such Co/InP nanocomposites can be gradually tuned from diamagnetism to ferromagnetism by increasing the deposition time of Co. Magnetic anisotropy of this Co/InP nanocomposite with magnetization easy axis along the axis of InP square channel was well realized by the competition between shape anisotropy and magnetocrystalline anisotropy. Such Co/InP nanocomposites with adjustable magnetism may have potential applications in future in the field of spin electronics.

**PACS**: 61.46. +w · 72.80.Tm · 81.05.Rm · 75.75. +a · 82.45.Aa

## Introduction

The fabrication and magnetic properties of magnetic nanomaterials or nanocomposites have been the center of attraction among researchers, due to their potential applications in high-density data storage devices, magneto-optical sensors, spintronic devices, and interesting fundamental physical phenomena [1-8]. Particularly, electrodeposition of magnetic nanoparticles, nanowires, and nanotubes in ordered nonmagnetic templates has attracted great attention because of its low cost, preferred yield of order magnetic nanomaterials, and size-adjustable properties [2,4,5,9-20]. The most popular template is anodic alumina oxide (AAO) membrane because of its uniform channel arrays and chemical inertness, which has been widely used for producing magnetic nanostructures, including cobalt ferrite nanodot arrays [13], Fe, Co, and Ni nanowires, nanotubes, and nanoparticles arrays [5,8,14-19], FeNi ferromagnetic alloy, CoPt nanotubes [9], and so on. The growth and magnetic properties of magnetic nanomaterials in AlN, MgO, polymer templates, and superlattice matrices were also reported [21-25]. Up to now, both theoretical and experimental works have focused mainly on insulation templates, while there has not been much study conducted on the growth of magnetic nanomaterials in semiconductor templates. Recently, electrodeposition of Fe, Co, Ni, and FeNi alloy into porous silicon semiconductor matrix has been studied [11,26-29]. It was found that the novel magnetization behaviors of these nanocomposite materials depended on deposits and matrices. For example, Granitzer et al. [29] found a new twofold switching of magnetic hysteresis curve in Ni/porous silicon composites. Therefore, electrochemical deposition of ferromagnetic metals into semiconductor templates and investigation of their magnetic properties may provide a new avenue for nanofabrication and have important applications ranging from magnetic sensing to the field of spin electronics [5,6,29].

In addition, owing to its direct band gap and enhanced nonlinear optical response, increasing interest has been focused on porous InP because of its potential applications in nanoscaled Schottky diodes, waveguides, solar cells, and for fabricating nanocomposite materials [30-34]. However, to our knowledge, there are no reports on the composite between porous InP matrix and magnetic materials. Furthermore, as a typical ferromagnetic materials, Co nanostructures, especially for granular Co, embedded in nonmagnetic matrices have been widely studied, where the matrices most focused were of three kinds: metallic matrices (Cu, Ag and Nb) [35-38]; ceramic matrices (Al_2_O_3 _and AlN) [5,23,39]; and polymeric matrices [22,24]. In this article, we report on the electrochemical deposition of Co into *n*-type porous InP semiconductor matrix based on organic solution of cobalt chloride, where the organic solution, i.e., ethanol solution, was applied to protect Co from oxidization. The structure and magnetic properties of such Co/InP nanocomposites were also investigated.

## Experiment details

**Figure 1:**
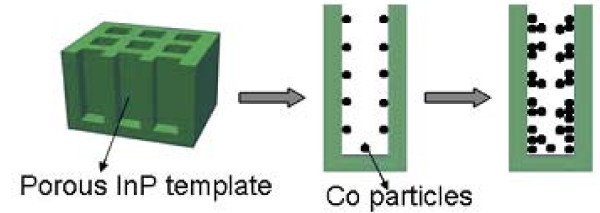
**Schematic of fabrication process of Co/InP nanocomposite structure**.

Co/InP magnetic semiconductor nanocomposites were fabricated by one-step electrodeposition of Co particles onto *n*-type porous InP templates. Figure 1 shows the schematic illustration of the fabrication of Co/InP composite structure. First, the *n*-type porous InP template was prepared by a two-step etching method [40]. The starting material was Sn-doped InP (>1 × 10^18 ^cm^-3^) wafer, which was first etched at a constant voltage of 8 V in 7.5% HCl aqueous solution for 30 s. Next, the specimen was immersed in a mixture of pure HCl and H_3_PO_4 _(HCl:H_3_PO_4 _= 1:3 v/v) for a few minutes to remove the top irregular layer to obtain *n*-type porous InP templates with uniform and square pore arrays. This was followed by electrochemical deposition of Co particles onto porous InP templates, performed using a three-electrode cell, employing a porous InP template as the working electrode and a graphite plate counter-electrode. The reference electrode was a saturated calomel electrode (SCE), isolated from the solution by a salt bridge. The deposition bath was 0.1 M/L CoCl_2 _ethanol solution, prepared by dissolving CoCl_2 _in ethanol. Before the deposition of Co, the porous InP template was immersed in the bath about 1 h to allow the solution completely wet the inner pore walls. The applied potential was kept at 2.0 V with respect to SCE. After the deposition of Co, the sample was cleaned by de-ionized water, dried in N_2 _atmosphere, and then kept in anhydrous ethanol. All the experiments were performed at room temperature.

The morphology of Co/InP nanocomposite structures was subsequently studied by field-emission scanning electron microscope (FE-SEM). The composition and crystallographic structure of samples were investigated by energy dispersive X-ray spectrometer (EDS) system attached to SEM and X-ray diffraction (XRD) with Cu Κ_α _radiation (λ = 1.54 Å). Physical property measurement system was applied to characterize magnetic properties of such Co/InP nanocomposites at 300 K with magnetic field sweeping from -15 to 15 KOe.

## Results and discussions

### Structure characterization of Co/InP nanocomposites

**Figure 2:**
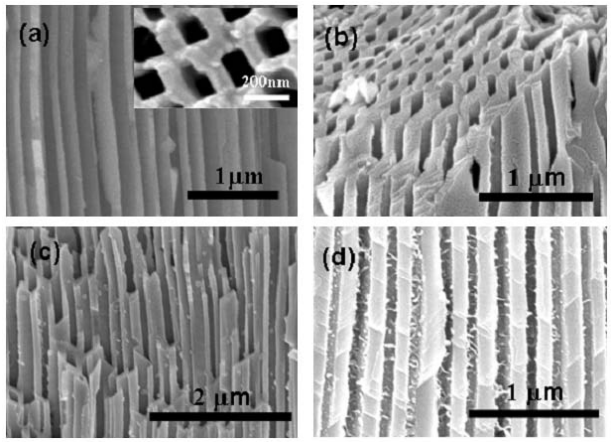
**FE-SEM images of the cross section of Co/InP nanocomposite structure with different deposition times of Co**: (a) 0 s, **(b) **30 s, **(c) **90 s, and **(d) **5 min.

Figure 2a shows the typical FE-SEM image of *n*-type porous InP template with nearly uniform and square pore arrays. In order to study the growth process of Co in porous InP semiconductor matrix, Co/InP nanocomposites with different deposition times were prepared. The cross-sectional morphologies of different samples are shown in Figure 2b, c, d. There is almost no Co in the inner channel wall of the InP matrix when the deposition time is 30 s, as shown in Figure 2b. When the deposition time increases to 90 s, it was found that a small amount of Co nanoparticles uniformly distribute on the whole inner channel walls of the InP template (Figure 2c). On further increasing the deposition time, the needle-shaped Co forms on the inner pore walls of InP as shown in Figure 2d. It is noted that Co particles prefer to uniformly distribute over the channel wall surface of the InP template than gather at the bottom of channel, which may result from conductivity of *n*-type porous InP template. In other words, the deposition of metallic Co particles may occur at any position of the pore sidewall surface of InP template (as shown by the schematic of Figure 1), which is different from the "bottom-up" growth mechanism in the insulation templates, such as AAO. Since the channel walls of the insulation templates are stable and nonconductive in the solution, the growth by electrodeposition is always from the bottom to the opening when a conductive layer is fabricated at the bottom side of the insulation channels [9]. Therefore, adjustable electrodepositions may be realized by tuning the conductivity and reactivity of such porous InP matrix, which may open up a new branch in the fabrication of nanocomposite materials. A detailed discussion for this is not given here because it is not the main concern for this article; similar studies in porous silicon matrix have been summarized by Ogata et al. [41].

**Figure 3:**
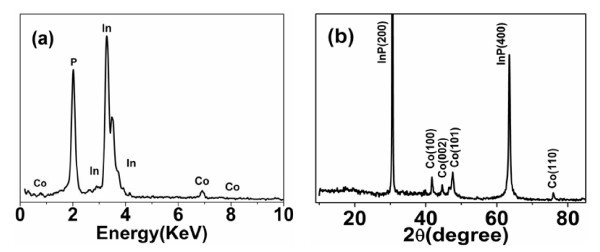
**The characterization of the Co/InP nanocomposite structure: **(a) **EDS spectrum and **(b) **XRD pattern**.

It is also noted that there is oxygen in the first product prepared by electrodeposition in aqueous solution of cobalt chloride under same conditions (this EDS spectrum is not illustrated in this article), which indicates that Co has been oxidized. Therefore, ethanol solution is chosen to fabricate Co/InP nanocomposites, the composition of which is analyzed by EDS as shown in Figure 3a, where only In, P, and Co exist (without the presence of oxygen), indicating that the pure Co nanoparticles have been successfully embedded in the porous InP semiconductor matrix and the ethanol solution effectively protects Co from oxidization. To further investigate the structure and composition of such Co/InP nanocomposites, the XRD pattern has been measured and shown in Figure 3b, where two strong diffraction peaks at 2θ = 30.52° and 63.41° are, respectively, identified as (200) and (400) of the porous InP template consistent with the previous results [34,40]. The other four peaks at 2θ = 41.59°, 44.26°, 47.39°, and 75.89° correspond to hexagonal Co (100), (002), (101), and (110), respectively. This further confirms that the obtained sample is that of Co/InP nanocomposites.

### Magnetic properties of Co/InP nanocomposites

**Figure 4:**
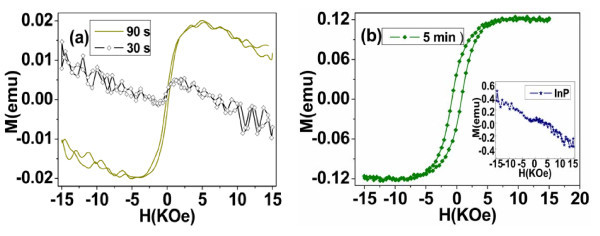
**Field-dependent magnetization curves (*M-H*) of the Co/InP nanocomposite structure, where the magnetic field is applied perpendicular to the surface of the InP template with different deposition times of Co**: **(a) **30 s (square) and 90 s (solid line), **(b) **5 min (square), the inset shows the magnetization curve of the *n*-type porous InP template (square).

Figure 4 shows field-dependent magnetization (*M-H*) curves of such Co/InP nanocomposites, where the applied magnetic field is perpendicular to the surface of the InP template or parallel to the axis of InP channel. For the deposition time of 30 s, the Co/InP nanocomposite presents diamagnetism as shown in Figure 4a, which is ascribed to the complete diamagnetism of *n*-type porous InP template according to the above SEM analysis and the *M-H *curve of pure InP (Figure 4b). While weak ferromagnetism is detected for the sample with the deposition time of 90 s (Figure 4a), with the deposition time of 5 min, the Co/InP nanocomposite exhibits visible hysteresis loop as shown in Figure 4b. This indicates that the Co particles embedded in the InP matrix dominate the magnetic behavior of this Co/InP nanocomposite when the content of Co gradually increases due to the strong ferromagnetism of Co. In a word, the magnetism of such Co/InP magnetic semiconductor nanocomposite is completely determined by the deposition time of Co. The exhibited ferromagnetism under the room temperature, originating from the Co particles embedded in the *n*-type porous InP matrix, is different from that of the superparamagnetism of Co particles in Cu and dendrimer matrix [22,36].

**Figure 5:**
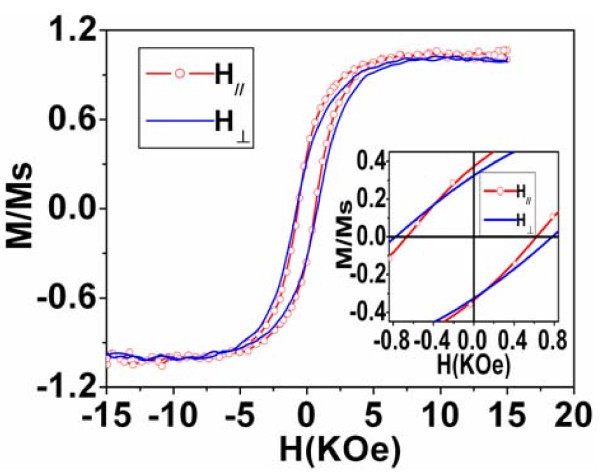
**Magnetic hysteresis loops of the Co/InP nanocomposite structure when the magnetic filed is applied both parallel (circle) and perpendicular (solid line) to the surface of the InP template with deposition time of 5 min**. Inset is the magnification curve.

Figure 5 shows magnetic hysteresis loops of Co/InP composite structure with the deposition time of 5 min for both perpendicular and parallel orientations, where *H*_// _and *H*_⊥ _represent the field applied perpendicular and parallel to the surface of the InP template, respectively. Typical coercivities with *H*_c⊥ _= 775 Oe and *H*c_// _= 644 Oe are clearly found in the inset of Figure 5, indicating the enhanced coercivity compared with that of the bulk Co (10 Oe). The relatively larger coercivity in perpendicular orientation suggests weak anisotropy of the system, i.e., magnetization easy axis is perpendicular to the surface of InP template. This magnetic anisotropy of the system is determined by the relatively strong-shape anisotropy of Co nanoparticle arrays embedded in the porous InP matrix compared with the magnetocrystalline anisotropy of hexagonal Co particle. Furthermore, both magnetization curves for perpendicular and parallel are sheared as shown in Figure 5, indicating the existence of inter-particle interactions, which is also manifested by the low squareness ratios, (*M_r_*/*M_s_*)_⊥ _= 0.34 and (*M_r_*/*M_s_*)_// _= 0.36. Similar sheared hysteresis loops were also found in Co/ZrO_2_, Co/AAO, and Ni/AAO nanocomposite materials [2,14,17]. In brief, magnetic anisotropy in the Co/InP nanocomposite structure with easy axis perpendicular to the surface of InP matrix is compatible with that of typical magnetic nanostructures such as nanowires and nanotubes [8,14,16,20,21,29], i.e., the magnetization easy axis is along the long axis of nanostructures, which is the result of the competition between the dominant shape anisotropy and magnetocrystalline anisotropy.

## Conclusion

We have reported in this article a novel Co/porous InP magnetic semiconductor nanocomposite based on electrochemical deposition technique in ethanol solution of cobalt chloride. The ethanol solution effectively protects Co from oxidization, as confirmed by the XRD and EDS analyses. Granular Co prefers to uniformly distribute over the channel walls of the InP templates, which is different from the "bottom-up" mechanism of ceramic matrix and thereby may provide a new avenue for nanofabrication. With the increasing deposition time of Co, the size or content of granular Co embedded in the InP template increases, and the magnetic behavior of such Co/InP nanocomposites shows gradual change from diamagnetism to ferromagnetism. The comparison of shape anisotropy effects to magnetocrystalline anisotropy effects helps one to explain the magnetic anisotropy of this novel Co/InP magnetic semiconductor nanocomposite, which may lead to new applications in the field of spin electronics.

## Abbreviations

AAO: anodic alumina oxide; EDS: energy dispersive X-ray spectrometer; FE-SEM: field emission scanning electron microscope; *M-H*: field-dependent magnetization; SCE: saturated calomel electrode; XRD: X-ray diffraction.

## Competing interests

The authors declare that they have no competing interests.

## Authors' contributions

TZ participated in the design of the study, carried out the experiments, performed the statistical analysis, as well as drafted the manuscript. DDC participated in the design of the study, carried out the experiments, and performed the statistical analysis. MJZ participated in the design of the study, provided the theoretical and experimental guidance, performed the statistical analysis, and revised the manuscript. LM participated in the design of experimental section and offered her the help in experiments. WZS gave his help in the setting up of experimental apparatus.
